# Mental health impact of the COVID‐19 pandemic on patients with neurodegenerative diseases and perceived family caregiver burden in Lima, Peru

**DOI:** 10.1002/brb3.3361

**Published:** 2024-01-02

**Authors:** Monica M. Diaz, Maisie Bailey, Bettsie Garcia, Ximena R. Aguilar, Danilo Coronel Sanchez

**Affiliations:** ^1^ Department of Neurology University of North Carolina at Chapel Hill Chapel Hill North Carolina USA; ^2^ School of Medicine Oregon Health Sciences University Portland Oregon USA; ^3^ Department of Neurodegenerative Diseases Instituto Nacional de Ciencias Neurologicas Peru Lima Peru

**Keywords:** caregiver burden, COVID‐19, dementia, Latin America, Parkinson's disease, Peru

## Abstract

**Background:**

Neurodegenerative diseases lead to difficulties with functional activities. In Peru, most caregivers are family members. Little is known about the COVID‐19 pandemic's effect on caregivers in Peru.

**Methods:**

This was a cross‐sectional, prospective study of family caregivers of dependent patients with dementia or Parkinson's Disease in Lima, Peru. A caregiver burden and mental health questionnaire was administered to the caregiver.

**Results:**

We enrolled 48 caregivers (65% females, mean ± SD age 49.0 ± 12.3 years); 70% of patients had dementia. Nearly 40% of caregivers reported having full‐time jobs, and 82% felt overwhelmed with almost 75% dedicating more time to caregiving during the pandemic. Caregivers perceived patients felt lonelier (52%), had an increase in hallucinations (50%), or forgetfulness (71%) compared to pre‐pandemic.

**Conclusions:**

Our study highlights that perceived caregiver burden and patient behavioral symptoms may have been exacerbated during the pandemic. In countries such as Peru, more caregiving resources and interventions are needed.

## 1 INTRODUCTION

Progressive neurodegenerative diseases, such as dementia and Parkinson's disease (PD), may lead to difficulties completing activities of daily living (ADLs) (Beaton et al., 2022; Gale et al., 2018). Informal caregivers of patients with neurodegenerative diseases, mostly family or household members, often manage the day‐to‐day needs of patients with these neurodegenerative conditions, including toileting, shopping, cooking, medication management, among others (Novais et al., 2017). Informal caregiver burden is often due to emotional and physical exhaustion, social isolation, or stigma, particularly in low‐to‐middle‐income countries (LMICs) with limited caregiving resources (Fam et al., 2019; Nguyen et al., 2021).

The COVID‐19 pandemic, caused by the novel coronavirus SARS‐CoV‐2, led to unprecedented challenges in many LMICs, particularly for vulnerable populations such as older adults and those with neurodegenerative diseases. People living with dementia are at high risk of depression, isolation, and behavioral changes, which may have been exacerbated by the COVID‐19 pandemic (Nakanishi et al., 2023). Prior studies have demonstrated that caregiver burden increased during the pandemic among informal caregivers of patients with PD (Hattori et al., 2023; Suzuki et al., 2021) and dementia in studies in India (Mukherjee et al., 2022), Thailand (Wongmek et al., 2023), Hong Kong (Fong et al., 2021), and across the United States (Mitchell et al., 2023; Yan et al., 2023). Caregiver burden increased in many regions of the world due to factors including aggravation of patient's symptoms, increased stress and anxiety, and needing to spend more hours at home given patients were less able to go out of the home during the pandemic (Carbone et al., 2021; Hattori et al., 2023). However, no studies have assessed caregiver burden in Peru during the pandemic.

Peru had the highest mortality rate in the world due to COVID‐19 despite an initially strict enforced lockdown, with 665.84 per 100,000 population up until March 2023 (Anon, n.d.a, n.d.b) outweighing that of the United States (thensecond highest in the world (341.11 per 100,000 population) and Chile (336.22 per 100,000) (Anon, n.d.b). Lack of oxygen, hospitals, ICU beds, and trained ICU healthcare professionals is thought to have led to the high mortality rate (Schwalb & Seas, 2021). Due to high population density and a fragile healthcare system before the pandemic, Peru faced unique challenges in protecting vulnerable older adults (Garcia et al., 2020; Giraldo, 2020). The impact that the pandemic had during the lockdown period and when social distancing was enforced in public places (March 2020–October 2022) (Anon, n.d.c) on caregivers of patients with neurodegenerative diseases is unknown in Peru, as in much of Latin America. Depressive symptoms and increases in behavioral changes have been reported in patients with dementia in studies from Argentina and Brazil (Azevedo et al., 2021; Sorbara et al., 2021), but little is known about the mental health and behavioral symptoms of patients with neurodegenerative diseases in Peru.

We hypothesized that the COVID‐19 pandemic in Peru would increase perceived caregiver burden and mental health symptoms (including depression and anxiety) both among patients and family caregivers with neurodegenerative diseases. The aims of the present study were to determine the impact on patient mental health symptoms from the perspective of the caregiver and caregiver burden during the COVID‐19 pandemic in a single center in Lima, the capital city of Peru.

## 2 METHODS

### 2.1 Study participants

This was a prospective study of patients who regularly attended and received neurological care in the Department of Neurodegenerative Diseases in the National Institute for Neurological Sciences (*Instituto Nacional de Ciencias Neurologicas*), a specialized neurology public hospital and a tertiary referral center located in Lima, Peru. We enrolled participants from August 2021 until February 2023. We included family caregivers who identified themselves as the primary informal caregiver of a patient with dementia, PD, or other chronic neurodegenerative diseases. Inclusion criteria were (1) the caregiver must have been at least 18 years of age at the time of enrollment and be able to provide verbal informed consent and (2) the patient had to be dependent on the caregiver for ADLs at least half of the time as determined by the treating neurologist. We excluded caregivers of patients who were dependent on ADLs less than half the time.

### 2.2 Study procedures

All participants were referred to the study by the treating neurologist if the participant met the inclusion criteria. A study research assistant (Ximena R. Aguilar) then contacted the caregiver by phone and completed the verbal telephone informed consent process. After participants (caregivers) provided verbal informed consent, a questionnaire was administered to participants by telephone.

### 2.3 Study measures

There were no validated scales in the literature that measured the intended outcomes. Therefore, we developed a 53‐item un‐scored questionnaire asking the caregiver questions that we felt were relevant to assess patients’ and caregivers’ perceived behavioral and mental health symptoms and caregiver burden compared with prior to the pandemic. An “increase” in a symptom was defined as a self‐reported increase in the symptom being assessed compared with prior to the COVID‐19 pandemic from the perspective of the caregiver. The questionnaire was not based on an aggregate score, but rather individual items (yes/no) were evaluated and compared with the outcomes of interest.

### 2.4 Patient characteristics and perceived behavioral changes

Patient demographics and characteristics were obtained from chart review or from the patient's treating neurologist. The patient's functional activities of daily living were assessed by asking the caregiver what types of activities they must do or help the patient at least half of the time, including shopping, medication management, going outside of the home, and cooking. The patient's perceived behavioral characteristics from the perspective of the caregiver were assessed, including sadness, loneliness, contact with family members, anxiety, among others. Patient COVID‐19 infection and vaccination questions were asked (see Supporting Information for complete questionnaire translated from Spanish).

### 2.5 Caregiver characteristics, burden, and mental health symptoms

Caregiver demographics were self‐reported, including questions on if they are currently working for compensation, socioeconomic characteristics, and remunerated additional caregiver support. Household characteristics were then obtained from the caregiver, such as the number of residents in the patients’ household, if other informal caregivers care for the patient, among others. We assessed the level of caregiver burden during the COVID‐19 pandemic by administering a questionnaire adapted and modified to query changes that occurred compared with prior to the pandemic from a scale previously translated and validated into Spanish, the *Zarit Caregiver Burden Scale* (Martin‐Carrasco et al., 2010). Two open‐ended questions (Supporting Information) were asked from the caregiver.

### 2.6 Ethical approval

We received ethical approval from the *Instituto Nacional de Ciencias Neurologicas* ethical review committee (IRB# N 115‐2021‐DG‐INCN) and the University of North Carolina at Chapel Hill internal review board (IRB# 21–1718).

### 2.7 Statistical analyses

To determine the effect of the pandemic on perceived patient behavioral changes and mental health symptoms, as well as perceived caregiver burden, we conducted descriptive analyses for continuous variables (mean and standard deviation) and categorical variables (*n* and frequency). All variables on the questionnaire were categorical (yes/no) variables except for two open‐ended questions. We used unadjusted logistic regression analyses to compare groups with categorical variable outcomes of “increase/same patient perceived depression” or “worsening depression.” All relevant predictor variables including patient and caregiver demographics/characteristics were assessed to determine if there was a relationship between the outcome of interest and perceived patient depression. We did not adjust for any covariates in the model nor for multiple comparisons as these did not contribute clinically to the outcomes. The two open‐ended questions were analyzed for patterns or themes and described in Section 3. All analyses were conducted on JMP Pro 17.0.

## 3 RESULTS

### 3.1 Informal caregiver demographics and characteristics

We included 48 caregivers in the study with a mean ± standard deviation (SD) age of 49 ± 12.3 years, and more than 60% were female. Most had completed up to high school. More than half said they had a full‐time paid job other than caregiving (Table 1).

**TABLE 1:** Patient, caregiver, and household characteristics (N = 48).

	*n* (%) or mean (SD)
Caregiver demographics	
Age	49.0 (12.3)
Female sex	31 (64.6%)
Educational level	
High school degree completed	15/48 (31.3%)
Technical school but did not complete degree	13/48 (27.1%)
Technical school completed	10/48 (20.8%)
University completed	9/48 (18.8%)
Relationship to patient	
Daughter or son (adult‐aged)	28/47 (59.6%)
Spouse	10/47 (21.3%)
Brother or sister	4/47 (8.5%)
Full‐time job other than caregiving	25/47 (53.2%)

Patient demographics	
Age	73.8 (11.0)
Female sex	35 (73.0%)
Where does the patient live?	
In primary caregiver's home	24/48 (50%)
In another family member's home	17/48 (35.4%)
In patient's own home	7/48 (14.6%)

Patient diagnosis	
Dementia	34/48 (70.83%)
Parkinson's disease	9/48 (18.75%)
Other^a^	5 (10.4%)
Time since initial diagnosis (years)	4.22 (4.33)

Primary insurance coverage (patient)	
National health insurance (SIS)	19/47 (40.43%)
ESSALUD	18/47 (38.30%)
Uninsured	6/47 (12.77%)
Private insurance	2/47 (4.26%)

Home environment	
Number of people per household	4.78 (2.09)
Owner of household	
Caregiver	24/48 (50%)
Family member non‐caregiver	17/48 (35.42%)

Abbreviation: SIS: Seguro Integral de Salud (Comprehensive Health Insurance); ESSALUD: Seguro Social de Salud (Social Health Insurance)

^a^ Other included Huntington's disease (1), viral encephalitis (1), stroke (1), and diagnosis unknown but followed in neurodegenerative clinic (2).

### 3.2 Patient demographics, characteristics, and home environment

The mean age of patients was 73.8 (SD 11.0) years with almost 75% female patients. Half of the patients lived in their primary informal caregivers’ homes. The majority (70.8%) of participants had dementia, and almost 20% had PD. Surprisingly, 12.8% of patients were uninsured, despite having access to national health insurance for all Peruvian citizens. Nearly one‐third of patients had tested positive for COVID‐19 at the time of the telephone interview, and nearly all patients were fully vaccinated with a booster (Table 1).

### 3.3 COVID‐19 pandemic perceived effects on patients as reported by caregivers

Many caregivers had difficulties obtaining medications for patients (48%) and accessing doctor or healthcare visits during the pandemic (74%). A majority of patients were perceived to be lonelier (52%) and had an increase in anxiety (60%), agitation (43%), hallucinations (50%), and forgetfulness (71%) as perceived by their caregiver compared to before the pandemic (Table 2). We found that 45% had difficulty complying with mask‐wearing, and 45% were unaware of the COVID‐19 pandemic as reported by the caregiver.

**TABLE 2:** Questions assessing caregiver burden compared with prior to the COVID‐19 pandemic (N = 48).

Caregiver factors	*n* (%)

Type of ADL caregiver completes for a patient	
Shopping (food, clothing, other)	16/20 (80)
Medication management	17/20 (85)
Accompany patient outside of the house all the time	15/20 (75)
Prepare food/cooking	18/20 (90)

Caregiving characteristics during a pandemic	
Increase in caregiver burden	38/46 (82.6)
Mild	10/38 (26.3)
Moderate	17/38 (44.7)
Severe	10/38 (26.3)
Increase in dedicating more time to caregiving	34/46 (73.9)
Difficulty explaining the pandemic to the patient	22/46 (47.8)
Difficulty asking patient to wear a mask	21/46 (45.7)
Has the caregiver found the pandemic to be a difficult period?	29/45 (64.4)
Experienced difficulties accessing healthcare and doctor's visits for the patient during the pandemic	34/46 (73.9)

Abbreviation: ADL, activity of daily living.

When comparing patients who had an increase in depressive symptoms to those who had the same or worsening of depressive symptoms, younger caregivers were significantly more likely to report depressive symptoms in patients. An increase in perceived patient depression was significantly associated with an increase in agitation and aggression and an increase in hallucinations or delusions and insomnia as perceived by the caregiver compared to before the pandemic (Table 3).

**TABLE 3:** Factors associated with an increase in depressive symptoms among patients with Alzheimer's disease or Parkinson's disease in Lima, Peru, during the COVID‐19 pandemic (N = 48).

Demographics	Patient depression increase	Patient depression same or improved	*p*‐value

Caregiver			
Age	44.5 (11.17)	53.14 (12.41)	**.017**
Female sex	14 (58.3%)	15 (68.2%)	.489

Patient			
Age	73.21 (11.23)	73.55 (11.09)	.92
Female sex	19 (72.2%)	15 (68.2%)	.397
Unaware of pandemic	11 (45.8%)	10 (45.5%)	.979
Able to socially isolate	24 (100%)	20 (90.9%)	.185
No contact with others outside the home during the pandemic	10 (41.6%)	13 (59.1%)	.238
Did not stay in contact with family members during pandemic	2 (8.3%)	5 (22.7%)	.175
Difficulty obtaining medications	13 (54.2%)	7 (31.8%)	.127
More anxious	18 (75.0%)	11 (50.0%)	.079
More agitated or aggressive	15 (62.5%)	5 (22.7%)	**.007**
More arguments with the primary caregiver	9 (37.5%)	6 (27.3%)	.460
Increase in hallucinations or delusions	17 (70.8%)	7 (31.8%)	**.008**
Increase in memory loss	19 (79.2%)	15 (68.2%)	.397
More difficulty completing activities of daily living	18 (75.0%)	13 (59.1%)	.250
More insomnia	17 (70.8%)	6 (27.3%)	**.003**

Bold *p*‐values are *p*‐values that are significant (*p* < 0.05).

### 3.4 COVID‐19 pandemic effect on informal caregivers

Most caregivers in this study were responsible for several independent ADLs (Table 2). More than 80% of caregivers felt more caregiver burden compared with before the pandemic. Very few (4.25%) had a remunerated additional caregiver. Almost 75% had to dedicate more time to caregiving during the pandemic. When asked to explain why they felt burdened by caregiving, some of the responses included the following: having to increase caregiving of the patient more than previously, interference with their paid job, being unable to leave the home, and feeling isolated, as well as economic strain. One caregiver stated that due to a family member admitted in the hospital with COVID‐19, family members needed to divide the caregiving responsibilities.

## 4 DISCUSSION

We found that more prevalent factors among patients with an increase in depressive symptoms during the pandemic were younger caregivers and an increase in agitation, hallucinations/delusions, and insomnia/poor sleep. However, factors such as an increase in memory loss and less social contact were not associated with an increase in depressive symptoms during the pandemic. About 80% of caregivers identified with having more perceived caregiver burden during the pandemic compared to prior to the pandemic. We also found that many caregivers had a compensated job despite the patient requiring assistance with most ADLs, which may exacerbate caregiver burden. Interestingly, about 35% of the caregivers did not find the pandemic to be a difficult period, which may highlight that there may be some resiliency or variable home factors that may contribute to this finding.

To our knowledge, only four studies in Latin America have assessed family caregiver burden of patients living with dementia. A study from Argentina found that 47.2% of the caregivers had caregiver burden on the *Zarit Caregiver Burden Scale* (Sorbara et al., 2021), and another Argentinian study found that caregiver burden during the pandemic was high irrespective of patient dementia severity (Cohen et al., 2020). Two other studies from Brazil used a similar methodology to ours asking family caregiver participants to compare their current state to prior to the pandemic and also found that caregivers were more overwhelmed during the pandemic, particularly when caring for patients with more severe dementia (Azevedo et al., 2021; Penteado et al., 2020). These findings are similar to ours, except that we also included patients without dementia in addition to those with dementia. Other studies have assessed caregiver burden during the pandemic for other neurological disease. For example, one study from the United States of caregivers of patients with epilepsy found over half (58%) had caregiver burden reporting increased anxiety (65%) and stress (64%) during the pandemic (Viny et al., 2023). Another study of caregivers of patients with amyotrophic lateral sclerosis found that caregiver burden was higher during the pandemic likely due to the restriction of external household member help (Giusiano et al., 2022). Our findings demonstrate that elevated perceived caregiver burden during the pandemic are consistent with findings throughout Latin America in studies on dementia, as well as other neurological conditions.

Patients with neurodegenerative diseases, including PD or dementia, have higher mortality and morbidity after COVID‐19 infection (Hua et al., 2022; Kim et al., 2022), which may increase the responsibility felt by the patient's caregiver to prevent COVID‐19 transmission to the patient. Moreover, cognitive impairment among patients with dementia and physical limitations experienced by many patients with PD can impede the ability to comply with COVID‐19 safety measures, increasing the burden felt by caregivers, seen in prior studies (Budnick et al., 2021; Otobe et al., 2022). Our study found that many patients were unaware of the pandemic, and many had difficulty in complying with mandatory mask‐wearing in Peru, likely exacerbating the perceived caregiver burden. Other factors such as unfamiliar environments and social isolation may exacerbate aggression or agitation, hallucinations or delusions, cognitive impairment, or depressive symptoms experienced by the patient (Azevedo et al., 2021; Mukherjee et al., 2022), as was seen in our study with most patients having an exacerbation of behavioral or mental health symptoms as perceived by the caregiver. Moreover, dependence on a caregiver, particularly if the caregiver had COVID‐19 infection and had to quarantine away from the patient, places an additional burden on the caregiver and other family member caregivers.

We found that many patients experienced a delay in their medical care and had difficulty accessing their medical providers and medications. In one study in Peru of patients with dementia during the pandemic, patients had a significant increase in depression, agitation, abnormal motor activity, sleep disorders, and appetite changes assessed by various measures (Custodio et al., 2021). In another study in Peru, only about 10% of patients with dementia had an increase in neuropsychiatric symptoms once the lockdown had been lifted (Custodio et al., 2023). Although our study was conducted when social distancing was mandated, our results are consistent with those reported in other Peruvian studies. If patients were experiencing an increase in hallucinations, delusions, or depression, limited access to healthcare or medications may worsen these symptoms. These findings highlight the need to make telemedicine capabilities widely accessible in order to counter the limited healthcare and medication access that can exacerbate behavioral symptoms.

In our study, we found that about 80% of caregivers identified as being more overwhelmed during the pandemic compared to prior to the pandemic. Almost three‐quarters of the caregivers had to increase their caregiving responsibilities during the pandemic despite the majority having a full‐time paid job. Few studies have assessed the burden of caregiving responsibilities during the pandemic in LMICs. However, in high‐income countries, such as Japan, rates of caregiver burden have been reported at 31% (Hattori et al., 2023), while studies from Germany and Slovenia also found caregiver burden increase during the pandemic.Factors that exacerbated caregiver burden were an increase in caregiving and loss of support, multiple patient health problems, and increased care duration (Budnick et al., 2021; Hvalič‐Touzery et al., 2022). In LMICs, such as in India, caregiver burden was exacerbated by feelings of helplessness and new situations brought about by lockdown and the pandemic (Mukherjee et al., 2022). In our study, we found that caregivers mostly expressed feeling burdened due to social isolation, increase in caregiving responsibilities, and needing to share caregiving among several family members. We found that some caregiver participants expressed feeling isolated and that they did not have sufficient family support. With the added burden of needing to protect the patient from COVID‐19 infection, this placed an additional stressor on many caregivers, as was seen in other studies (Grycuk et al., 2022; Manca et al., 2022; Pongan et al., 2021). This demonstrates that increasing caregiver support in LMICs is needed, particularly in resource‐limited settings where the primary caregivers are often informal caregivers with no formalized training.

Unpaid dementia caregiving is estimated to cost $339.5 billion in 2022, which is expected to have been exacerbated by the COVID‐19 pandemic with increases in emotional distress and negative physician and mental health outcomes of unpaid caregivers worldwide (Anon, 2023; Ibáñez et al., 2021). Prior to the pandemic, a lack of support resources, such as a lack of rehabilitation programs, long‐term care facilities, and accessible buildings and infrastructure, and a lack of available dementia medications make it difficult for people with neurodegenerative diseases to have a life with higher quality (Fuhs et al., 2018; Parra et al., 2021). These existing limitations were likely further exacerbated by the pandemic as demonstrated by our study.

One potential solution to counter limited healthcare during the pandemic was offering telehealth and telemedicine services. For example, one study from Italy implemented a telehealth model for providing emotional support to informal caregivers of patients with dementia during the pandemic, which helped improve their mood (Rotondo et al., 2022). However, limitations exist to implementing telemedicine, including unstable access to the internet and unfamiliarity with technology in older populations (Camacho‐Leon et al., 2022; Miele et al., 2020). LMICs, such as Peru, would benefit from increasing access to telehealth technologies in rural areas of the country to prevent exacerbation of neurodegenerative conditions. Caregiver training programs and other interventions to help reduce caregiver stress have been piloted. One study in Italy demonstrated reductions in mental health symptoms among caregivers (Rotondo et al., 2022) and increased resilience and well‐being of both patients with dementia and their caregivers in another study (Lai et al., 2020) after implementing telehealth services. Using culturally adapted caregiver support interventions, including educational sessions on caregiving, may help improve caregiver burden in LMICs (Hinton et al., 2020). Culturally adapted caregiver resources, including in‐person or online support groups and other community initiatives (Masoud et al., 2021) and mental health support, are needed in Peru to help ameliorate symptoms of anxiety, depression, and caregiver burden.

Our study has limitations. First, the data collection occurred once the lockdown had been lifted (June 2020). However, in Peru, mandated mask‐wearing in public places and social distancing was required until October 2022. Therefore, the majority of participants were enrolled when social distancing was mandated. Our study reflects that although the lockdown may have been lifted, there was a high caregiver burden and patient behavioral symptoms. Next, this is a small cohort of caregivers of patients attending a specialized public neurology hospital in an urban area and thus may not be representative of more rural areas nor generalizable throughout the country nor to other LMICs nor high‐income countries. There was potential for selection bias, but treating neurologists invited all family caregivers who were felt to meet inclusion criteria. Next, we did not administer formal validated questionnaires to caregivers or patients. Therefore, we only have reports of subjective symptomology of anxiety, depression, and subjective cognitive decline from the caregivers’ perspective. Moreover, we are unable to establish any causal relationships with the logistic regression analyses performed. Lastly, we did not have pre‐ and post‐pandemic assessments for comparison which may lead to recall bias. Thus, our findings may not be comparable to those studies that had pre‐ and post‐pandemic assessments.

## 5 CONCLUSIONS

Our study found that caregiver burden was elevated and that caregivers felt more anxiety and depressive symptoms compared to before the pandemic. Our study sought to provide a comprehensive understanding of the challenges faced by caregivers during the pandemic which may help provide solutions for improving caregiver resources in Peru. We believe our study sheds important light on the caregivers’ perception of the patient's condition and how it affects their caregiver burden. This is the only study to have assessed the caregiver burden of patients with neurodegenerative diseases in Peru during the pandemic, and one of the few in Latin America.

Future studies may determine participants who did not find the pandemic to be a difficult period the reasons for this through in‐depth interviewing in order to determine which factors may lead to caregiver resiliency. Determinining the extent of economic loss due to caregiving is crucial for future studies. By examining the impact of COVID‐19 on Peruvians with neurodegenerative disease and their caregivers, this study has identified targets for the Ministry of Health and other organizations to improve mental health and community resources for caregivers of patients with neurodegenerative diseases, particularly during challenging times such as in the COVID‐19 pandemic. Moreover, our findings may serve to help develop targeted interventions and support systems for caregivers in Peru and throughout many LMICs.

## AUTHOR CONTRIBUTIONS

**Monica M. Diaz**: Conceptualization; investigation; writing–original draft; methodology; visualization; writing–review and editing; supervision. **Maisie Bailey**: Writing–original draft; methodology; data curation. **Bettsie Garcia**: Methodology; writing–review and editing; data curation. **Ximena R. Aguilar**: Data curation; writing–original draft; methodology. **Danilo Coronel Sanchez**: Conceptualization; investigation; writing–original draft; methodology; writing–review and editing; data curation.

## CONFLICT OF INTEREST STATEMENT

The authors declare no conflicts of interest.

### PEER REVIEW

The peer review history for this article is available at https://publons.com/publon/10.1002/brb3.3361.

## Supporting information

Supplemental File I

## Data Availability

Data will be made available upon request from the corresponding author.
