# Supplementary material for: Mental health impact of the COVID‐19 pandemic on patients with neurodegenerative diseases and perceived family caregiver burden in Lima, Peru
Source: Brain Behav. 2024 Jan 2;14(1):e3361. doi: 10.1002/brb3.3361 (PMC10761328; doi:10.1002/brb3.3361)
Supplement: Supplementary file 1 — Supplemental File I [file BRB3-14-e3361-s001.docx]

**Supplementary File I**

**Caregiver Burden and Perception of Patient Symptoms Questionnaire** (Translated to English from Spanish version)

1. **Patient Demographics & Characteristics** (obtained from chart review)
2. Age
3. Sex
4. Patient’s diagnosis
5. Year of symptom onset
6. Who diagnosed the patient? (Neurologist/geriatrician/psychiatrist/other)
7. Type of insurance the patient has (SIS, ESSALUD, private insurance, no insurance)
8. **Caregiver Demographics and Household Characteristics** (addressed to caregiver)
9. Age
10. Sex
11. Relationship with the patient (parent, spouse, son or daughter, sibling, other relationship)
12. What was your highest level of education completed?
13. Are you currently employed or do you work for compensation?
    1. If yes, what is your current occupation?
    2. How many hours per day do you work at this job?
14. Who in the household is the head of household *(“principal sostenedor de hogar” in Spanish*)?
    1. Head of household’s current occupation
15. Do you also receive support from a remunerated caregiver in the home? / Is the patient cared for by a remunerated caregiver?
    1. If yes, was the remunerated caregiver’s time reduced during the pandemic?
16. **Household Characteristics** (addressed to caregiver)
17. How many people live in the patient’s home where their care is provided?
18. Where does she patient live? (caregiver’s home, home of another family member, other)
19. In what community did the patient quarantine during the pandemic (district, city, region)?
20. Are you the sole caregiver?
    1. If no, who else cares for the patient in the family? (parent, spouse, son or daughter, sibling, other relationship)
    2. Prior to the pandemic, did you have help from these additional caregivers/family members?
    3. Has extra caregiving support from other family members or friends been interrupted during the pandemic?
21. **Functional Activities of Daily Living** (addressed to caregiver): “What type of activities do you have to do for or help the patient all of the time?”
22. Shopping (groceries, clothing, home goods)
23. Help with medications or giving them their medications
24. Accompany them outside of the home
25. Cook or prepare food
26. **Patient Behavioral Characteristics** – *“Please answer the following questions based on how you perceive or think that the patient is doing since March 2020”:*
27. Does the patient understand what is going on with COVID-19 and the pandemic?
28. Is the patient worried about COVID-19 or the pandemic?
29. Did the patient quarantine during the mandatory quarantine?
30. Does the patient observe social distancing practices?
31. Does the patient have direct contact with people in the family who live OUTSIDE the patient’s residence since March 2020?
32. Has the patient seen or spoken with by phone family members or friends by telephone or video call since March 2020?
33. Is the patient exercising MORE or LESS then prior to March 2020 (ex. Walking inside the home for exercise, going up and down the stairs, other at-home exercise programs)?
34. Has the patient had difficulties obtaining their medications since March 2020?
35. Have you had more difficulties obtaining doctor’s appointments for the patient since March 2020?
36. Has the patient accepted these social changes, including social distancing?
37. Do you perceive the patient to been more sad compared with prior to March 2020?
    1. If yes, would you say it is mild, moderate or severe sadness?
       1. *Mild is described as 1-2 times per month; moderate 1-2 times per week; severe more than once per day*
38. Has the patient complained of feeling lonely?
    1. If yes, would you say it is mild, moderate or severe loneliness?
       1. *Mild is described as 1-2 times per month; moderate 1-2 times per week; severe more than once per day*
39. Do you perceive that the patient is more anxious compared with prior to March 2020?
    1. If yes, would you say it is mild, moderate or severe anxiety?
       1. *Mild is described as 1-2 times per month; moderate 1-2 times per week; severe more than once per day*
40. Do you perceive that the patient is more agitated or aggressive compared with prior to March 2020?
    1. If yes, would you say it is mild, moderate or severe aggression?
       1. *Mild is described as 1-2 times per month; moderate 1-2 times per week; severe more than once per day*
41. Is the patient having more arguments or conflicts with you compared with prior to March 2020?
    1. If yes, would you say it is mild, moderate or severe conflicts?
       1. *Mild is described as 1-2 times per month; moderate 1-2 times per week; severe more than once per day*
42. Has the patient had more paranoia (i.e. for example, thinking that someone has robbed them or that the house where they live is not their home) or hallucinations (i.e. see or hear something that is not there) compared with prior to March 2020?
    1. If yes, would you say it is mild, moderate or severe conflicts?
       1. *Mild is described as 1-2 times per month; moderate 1-2 times per week; severe more than once per day*
43. From your perspective is the patient having more memory loss or trouble remembering things compared with prior to March 2020?
    1. If yes, would you say it is mild, moderate or severe conflicts?
       1. *Mild is described as 1-2 times per month; moderate 1-2 times per week; severe more than once per day*
44. Is the patient having more difficulty with activities such as dressing themselves, bathing, or toileting compared with prior to March 2020?
    1. If yes, would you say it is mild, moderate or severe conflicts?
       1. *Mild is described as 1-2 times per month; moderate 1-2 times per week; severe more than once per day*
45. Has the patient had more difficulty falling asleep or staying asleep compared with prior to March 2020?
    1. If yes, would you say it is mild, moderate or severe conflicts?
       1. *Mild is described as 1-2 times per month; moderate 1-2 times per week; severe more than once per day*
46. **Caregiver Burden questions-** *“Please answer the following questions comparing how you currently feel to how you felt before the pandemic started in March 2020.”*
47. Do you feel as if you are dedicating more time to have to care for the patient compared with prior to March 2020?
    1. If yes, please rate: rarely, sometimes, frequently, or almost always
48. Have you had difficulty explaining and making the patient understand why they need to social distance or about the pandemic?
49. Have you felt like you have more responsibilities now with caregiving compared with prior to the pandemic?
    1. If yes, please rate: rarely, sometimes, frequently, or almost always
50. Have you had difficulties making the patient understand that they must wear a mask in outdoor spaces?
    1. If yes, please rate: rarely, sometimes, frequently, or almost always
51. Have you found the pandemic (especially the quarantine) to be difficult?
    1. If yes, rarely, sometimes, frequently, or almost always
    2. If yes, please describe what has been difficult for you? (*open-ended question)*
52. Have you felt more sad compared with prior to March 2020?
    1. If yes, rarely, sometimes, frequently, or almost always
53. Have you felt more anxious compared with prior to March 2020?
54. **COVID-19-related questions**
55. Does the patient have or have they had symptoms of COVID-19 (cough, fever, shortness of breath or others)?
    1. If yes, was the patient tested for COVID-19?
    2. If yes, what was the test result?
    3. If yes to 40, were they hospitalized?
56. Have you (the caregiver) ever tested positive for COVID-19?
    1. If yes, were you (the caregiver) hospitalized for COVID-19?
57. Did any family member that lives in the same household as the patient test positive for COVID-19?
    1. If yes, was the family member hospitalized?
58. Describe more details of the situations in #40-42, if necessary
59. Is the patient vaccinated?
    1. If yes, what vaccine did they receive?
    2. If yes, how many vaccine doses did they receive?
60. Are you (the caregiver) vaccinated?
    1. If yes, what vaccine did you receive?
    2. If yes, how many doses of vaccine did you receive?
